# High confidence HIF-1 target genes for quantifying the activation of the HIF-1-mediated hypoxia response

**DOI:** 10.17912/micropub.biology.002304

**Published:** 2026-09-15

**Authors:** Camille AH Desnos, Aura A Tamez Gonzalez, Jeremy M Van Raamsdonk

**Affiliations:** 1 Neurology and Neurosurgery, McGill University, Montreal, QC, Canada; 2 Metabolic Disorders and Complications (MeDiC), Research Institute of the McGill University Health Centre; 3 Neurology and Neurosurgery, McGill University, Montreal, Quebec, Canada

## Abstract

The hypoxia-inducible factor 1 ( HIF-1 )-mediated hypoxia response is an evolutionarily conserved stress response that enables adaptation to conditions of low oxygen and mitochondrial dysfunction. This pathway has been shown to affect both stress resistance and longevity. HIF-1 activity is regulated by EGL-9 , VHL-1 , and RHY-1 and induces the expression of genes involved in metabolism and cellular resilience. To find genes that can be used to monitor HIF-1 activation, we compared HIF-1 modulated genes identified by multiple RNA-seq experiments and HIF-1 target genes identified by ChIP-seq experiments. We identified high-confidence HIF-1 target genes that provide robust markers of pathway activation.

**Figure 1. Identification of high confidence HIF-1 target genes:** **(A)** Overview of HIF-1 mediated hypoxia pathway. Under normal oxygen conditions (normoxia) HIF-1 is hydroxylated by EGL-9. Hydroxylated HIF-1 is recognized and ubiquitinated by VHL-1 (Ub = ubiquitin groups). Ubiquitinated HIF-1 is degraded by the proteasome. Under hypoxic conditions, EGL-9 cannot hydroxylate HIF-1. As a result, HIF-1 travels to the nucleus to change gene expression with AHA-1. **(B)** Weighted Venn diagram showing overlap of HIF-1 modulated genes identified by Shen et al., Feng et al. and Vora et al. using RNA-seq. **(C)** Expression of HIF-1 target genes in *nuo-6* and *nuo-6;hif-1* mutants from RNA-seq data. **(D)** Heat map showing expression of top HIF-1 modulated genes in a panel of nine long-lived mutants. Expression is indicated as a percentage of wild-type expression. The heatmap was generated using Morpheus: https://software.broadinstitute.org/morpheus/ The greatest number of HIF-1 modulated genes are activated in *nuo-6* and *isp-1* mitochondrial mutants, while the fewest are activated in *osm-5* and *eat-2* mutants. RNA-seq data for the heat map is from Rudich et al. 2026, *eLife.* The RNA-seq data is available at NCBI GEO: GSE179825, GSE93724, GSE110984. **(E)** The expression levels of high confidence HIF-1 target genes in *isp-1* and *isp-1;hif-1* mutants using qPCR. Expression levels were normalized to *act-3* and then expressed as a percentage of wild-type expression. Statistical significance was determined using a two-way ANOVA with Šidák's multiple comparisons test in panel C and a one-way ANOVA with Dunnett's multiple comparisons test in panel E. ns = not significant, *p<0.05, **p<0.01, ***p<0.001, ****p<0.0001.

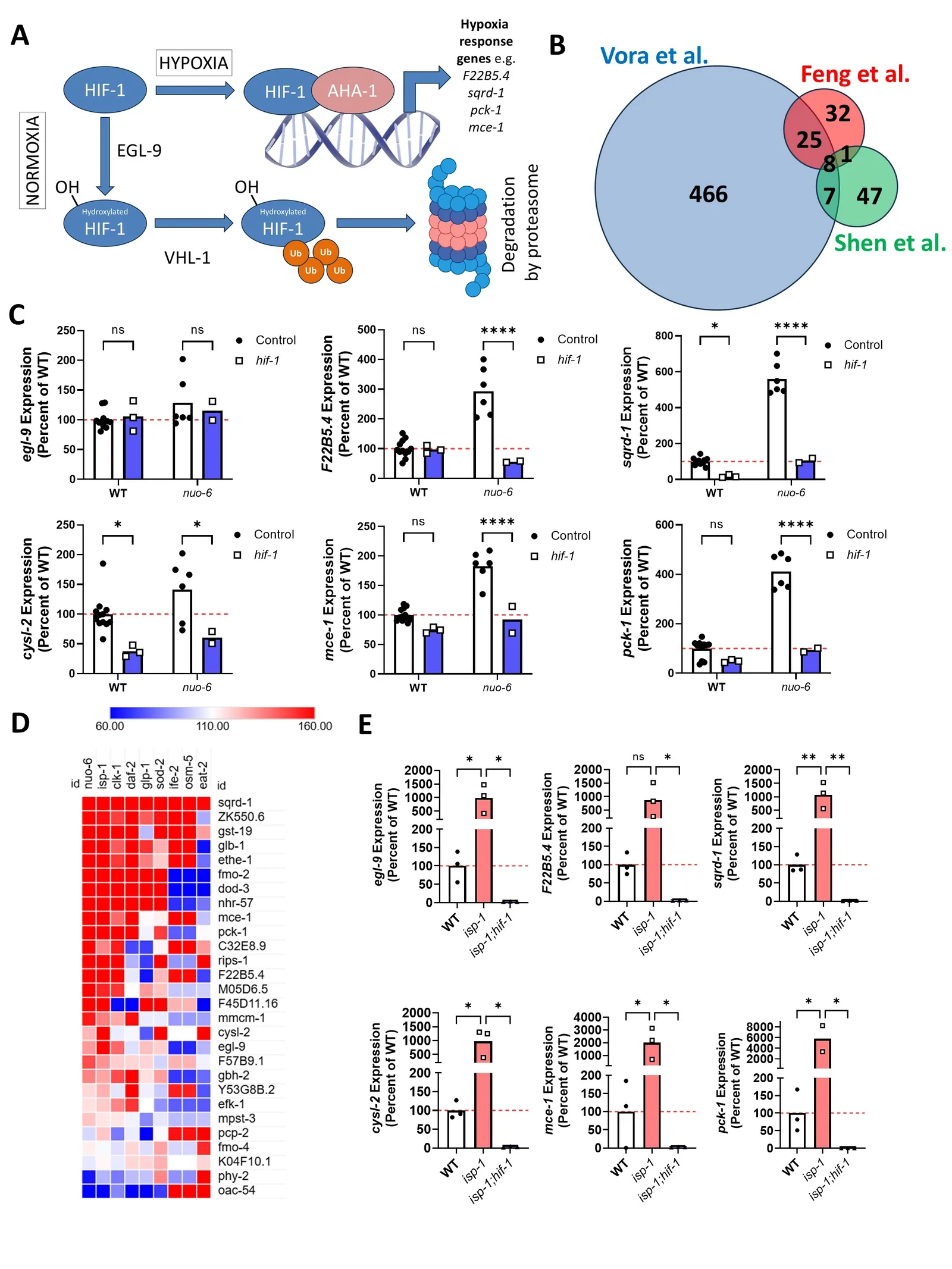



## Description

The HIF-1-mediated hypoxia pathway is a pathway of cellular resilience that is activated under conditions of low oxygen (hypoxia). The transcriptional changes for this pathway are mediated by the HIF-1 transcription factor, which acts in a heterodimer with AHA-1 . *hif-1* encodes the HIF-1 α subunit, which is sensitive to oxygen levels, while *aha-1* encodes the constitutively expressed HIF-1 β subunit. Under normoxic conditions, HIF-1 is hydroxylated by the prolyl hydroxylase EGL-9 (Epstein et al., 2001). Hydroxylated HIF-1 can then be recognized by the von Hippel-Lindau E3 ubiquitin ligase VHL-1 , resulting in ubiquitination and proteasomal degradation of HIF-1 (Bishop et al., 2004). Under hypoxic conditions (0.1% - 1% oxygen), there are insufficient levels of oxygen for EGL-9 to hydroxylate HIF-1 . As a result, HIF-1 is not hydroxylated and not marked for degradation by VHL-1 . Instead, HIF-1 is able to accumulate in the nucleus and induce changes in gene expression. HIF-1 can also escape degradation when either EGL-9 or VHL-1 are disrupted. In addition, HIF-1 degradation can be prevented by disrupting the RHY-1 (Regulator of hypoxia-inducible factor-1) transmembrane protein, which normally acts to reduce CYSL-1 levels and prevent CYSL-1 from binding to and inhibiting EGL-9 (Kruempel et al., 2020; Shen et al., 2006).

A number of previous studies have demonstrated a role for the HIF-1-mediated hypoxia response in lifespan determination. Increasing the levels or activation of HIF-1 either directly or through disruption of its negative regulators *vhl-1* , *egl-9* , or *rhy-1* , extends lifespan, indicating that activation of the hypoxia pathway can promote longevity (Kruempel et al., 2020; Leiser et al., 2015; Mehta et al., 2009; Zhang et al., 2009). In contrast, the results of studies examining the effects of *hif-1* disruption on lifespan have been more varied with some experiments showing increased, decreased, or unchanged lifespan depending on the exact conditions utilized (Leiser and Kaeberlein, 2010). HIF-1 has also been shown to contribute to the longevity of multiple long-lived mutants. HIF-1 is required for lifespan extension in the long-lived mitochondrial mutants *clk-1 , isp-1* and *nuo-6* (Lee et al., 2010; Wu et al., 2018), but dispensable for the enhanced longevity of *daf-2 , eat-2 , glp-1* and *osm-5* mutants (Lee et al., 2010; Soo et al., 2023). The HIF-1 target gene *fmo-2* is required for the lifespan extension induced by dietary restriction and by mild mitochondrial dysfunction (Leiser et al., 2015; Van Raamsdonk, 2026).

Multiple previous studies have used RNA sequencing (RNA-seq) to find genes that are differentially expressed following HIF-1 activation. Although the expression of these genes is dependent on HIF-1 , the effect of HIF-1 on gene expression could either be direct through HIF-1 binding to the promoter or indirect. As a result, we refer to these genes as HIF-1 modulated genes, though at least some of these genes are direct targets of HIF-1 .

Shen et al. identified genes that are upregulated during hypoxia with p<0.05 and fold change greater than or equal to 2 and not upregulated by hypoxia in *hif-1 ( ia4 )* mutants (Shen et al., 2005). Vora et al. identified genes that are upregulated in *egl-9 ( sa307 )* and *egl-9 ( sa307 ); hif-1 ( ia4 ):odIs131[ hif-1 ::gfp]* mutants compared to wild-type and *egl-9 ; hif-1 ( ia4 )* worms (Vora et al., 2022). Feng et al. identified genes that are induced by 1.6 fold more in wild-type worms compared to *hif-1 ( ia4 )* worms after a 2 hour exposure to hypoxia (Feng et al., 2024b). In a separate paper, Feng et al. identified genes upregulated in *vhl-1 ( ok161 ), rhy-1 ( ok1402 ), egl-9 ( sa307 )* and *swan-1 ( ok267 ); vhl-1 ( ok161 )* worms and made a list of genes commonly upregulated amongst all four strains (Feng et al., 2024a). Doering et al. identified genes upregulated by hypoxia in wild-type worms and *nhr-49* mutants but not in *hif-1* mutants (Doering et al., 2022). We previously compared gene expression between *isp-1* and *isp-1 ; hif-1* mutants, as the HIF-1-mediated hypoxia pathway has been found to be activated in *isp-1* mutants (Lee et al., 2010). For our study, HIF-1 modulated genes were selected as genes that are upregulated by at least 30% in *isp-1* worms compared to wild-type worms and decreased by at least 30% in *isp-1 ; hif-1* worms compared to *isp-1* worms.

In addition to these RNA-seq experiments, two studies have used Chromatin Immunoprecipitation sequencing (ChIP-seq) to find which gene promoters are bound by HIF-1 following activation. Feng et al. performed a ChIP-Seq experiment in which the *hif-1 a* isoform was labelled with a HA tag in the *egl-9 ( sa307 )* background and an anti-HA antibody was used for ChIP (Feng et al., 2024b). Vora et al. performed a ChIP-Seq experiment in which *egl-9 ( sa307 ); hif-1 ( ia4 );odIs131[ hif-1 ::gfp]* worms were compared to *hif-1 ( ia4 );odIs131[ hif-1 ::gfp]* worms (Vora et al., 2022).

To identify high confidence HIF-1 modulated genes, we initially compared the gene sets identified by Shen et al. (genes upregulated during hypoxia in a *hif-1-* dependent manner), Vora et al. (genes upregulated by *egl-9* mutation in a *hif-1-* dependent manner) and Feng et al. (genes induced 1.6 fold more in wild-type worms than *hif-1* worms after hypoxia) ( **Figure 1B** ). We found that there were 41 genes in at least two of these gene sets with just 8 genes found in all three. We then examined whether these 41 genes were identified in the other RNA-seq experiments and the published ChIP-Seq experiments. We found that three genes were identified in seven of the eight studies ( **Extended data** **Table S1** ) (The ChIP-seq experiment by Feng et al. only identified one of the 41 genes). This included *egl-9 , F22B5.4* and *sqrd-1 .* There were 8 more genes that were identified in six of the eight studies: *efk-1 , phy-2 , cysl-2 , gbh-2 , ethe-1 , mce-1 , ZK550.6 ,* and *pck-1* .

As we have previously used RNA sequencing to examine gene expression in *nuo-6* and *nuo-6 ; hif-1* worms, as well as wild-type and *hif-1* worms (Wu et al., 2018), we next examined the expression of a selection of these genes using this RNA-seq data. We found that disruption of *hif-1* significantly decreased the expression of *F22B5.4 , sqrd-1 , cysl-2 , mce-1* and *pck-1* , thereby providing additional support that these genes are dependent on HIF-1 ( **Figure 1C** ).

We recently examined gene expression in a panel of nine long-lived mutants representing multiple different pathways of lifespan extension (Rudich et al., 2026). To determine the extent to which the HIF-1-hypoxia pathway is activated in each of these strains, we examined the expression of HIF-1 modulated genes that were identified by at least four of the eight studies we examined. We found that *nuo-6* and *isp-1* showed the greatest activation of HIF-1 modulated genes, while *osm-5* and *eat-2* worms showed the least activation ( **Figure 1D** ). This is consistent with our observation that *nuo-6* and *isp-1* belong to a different longevity group than *osm-5* and *eat-2* worms, based on gene expression (Rudich et al., 2026). It is important to note that the expression of these HIF-1 modulated genes was examined at day 1 of adulthood. In addition to genotype, the transcriptional output of HIF-1 may vary by developmental stage, tissue, oxygen level, exposure duration and environmental stressors.

In order to facilitate other researchers quantifying the expression of HIF-1 target genes to measure activation of the HIF-1-hypoxia response, we designed primers to measure six of the high confidence HIF-1 target genes using quantitative RT-PCR (details on the binding location and amplicon size of the primers can be found in **Extended data File 1** ). These genes included *egl-9 , F22B5.4 , sqrd-1 , cysl-2 , mce-1* and *pck-1* . To test the function of these primers, we isolated RNA from *isp-1* and *isp-1 ; hif-1* worms with wild-type worms as control. We normalized the expression of each gene to *act-* 3, as our previous RNA-seq data showed that *act-3* levels are not significantly different between wild-type and *isp-1* worms. As anticipated, we found that the expression of all six selected HIF-1 target genes is upregulated in *isp-1* mutants and the upregulation of these genes in *isp-1* worms is completely prevented by the disruption of *hif-1* ( **Figure 1E** ). This confirms that the primers we designed can be used to effectively measure the expression levels of these HIF-1 target genes.

Overall, this work combined multiple previous RNA-seq and ChIP-seq studies to identify high confidence HIF-1 target genes that can be used to monitor the activation of the HIF-1-mediated hypoxia response. We also designed and validated qPCR primers to quantify these genes using quantitative RT-PCR.

## Methods

**Strains**

The following strains were used: N2 (wild-type), *isp-1 ( qm150 ), isp-1(qm150);hif-1(ia4)* . Strains were maintained at 20°C on NGM plates seeded with OP50 *E. coli* bacteria.

**RNA Isolation**

Worms were synchronized by a 4-hour limited time egg laying, collected at the pre-fertile young adult stage, and washed 3 times with M9. Trizol was then added and the samples were frozen in liquid nitrogen and kept at –80°C until RNA isolation. To extract the RNA, the pellet was thawed and re-frozen in liquid nitrogen 3 times, after which more Trizol was added to a total volume of 250 µl. This was followed by 3 vortex cycles consisting of 30s vortexing and 30s at room temperature. Samples were then left to sit at room temperature for 15 minutes and chloroform was added at 1:5 volume of Trizol. After shaking for 15 seconds, samples were left to sit at room temperature for 3 minutes. Then, samples were centrifuged at 12,000 *x g* for 15 minutes at 4°C, and the upper aqueous phase was transferred to a new tube. An equal volume of isopropanol was added, and it was left sitting on ice for 1 hour. After, samples were centrifuged at 12,000 *x g* for 20 minutes at 4°C, the supernatant was removed, and the pellet was washed with 75% ethanol. Then, samples were centrifuged at 12,000 *x g* for 10 minutes at 4°C, the ethanol was removed, and 100% ethanol was added before centrifuging at 12,000 *x g* for 3 minutes. The ethanol was removed and the pellet was air dried for 5 minutes until no traces of ethanol were found. The pellet was then dissolved in RNAse free double distilled water. RNA was isolated from 3 biological replicates.

**Quantitative PCR**

The samples underwent DNase I treatment to remove genomic DNA, using a DNase I, RNase-free kit (Thermo Scientific) following the manufacturer's instructions. Then, cDNA was synthesized using a High Capacity cDNA Reverse Transcription Kit (Applied Biosystems), according to the manufacturer's protocol. qPCR was performed using SYBR Green Master Mix (Applied Biosystems) in a Viia7 Real Time PCR System (Applied Biosystems). RNA levels were normalized to the expression of the *act-3* gene. After running the RT-PCR, expression levels were calculated as 2 ^-CT^ (CT is the cycle threshold, which is the number of cycles at which time the fluorescence crosses a specified threshold). The expression levels for each individual gene was then divided by the expression level of the control gene *act-3 .* Finally, the copies/copy *act-3* was divided by the average copies/copy *act-3* of the wild-type samples. Three biological replicates were performed.

**Primers**

The sequences for the primers used for qPCR are below:

**Table:** 

**Gene**	**Primer 1**	**Primer 2**	**Amplicon Size**
*egl-9*	TCGACAACCCTCCAAGAACA	GGCTTCTGATCACATGCTCG	115 bp
*F22B5.4*	ATGTTCCATCGCCAGCAAGA	ACGGCGGACAAGGAATTGATA	146 bp
*sqrd-1*	TGGTGGGTCATTACAGTCCAAA	TACATGGCCGATTACCCTGC	155 bp
*cysl-2*	TGGGTGGAATCTCGTCTGGA	CGTAGAGGGCGGTTGAAAGAT	132 bp
*mce-1*	TTCGCTGTCCACAAGAACCAT	TAACTTTTGCTCCGAGGCCC	131 bp
*pck-1*	CCACGTCCAGTTAAGCAAAAGG	AGCGAAGCACTTCTTTCCGA	147 bp
*act-3*	TGCGACATTGATATCCGTAAGG	GGTGGTTCCTCCGGAAAGAA	60 bp

## Reagents

Strains utilized:

**Table:** 

**Strain Name**	**Genotype**	**Source**
N2	wild-type	CGC
MQ887	*isp-1 ( qm150 ) *	Hekimi lab
JVR023	*isp-1 ( qm150 ); hif-1 ( ia4 ) *	Genetic cross

## Data Availability

Description: Extended Data Table S1. Resource Type: Dataset. DOI: https://doi.org/10.22002/5tjm5-nh142 Description: Extended Data File 1. Binding location and amplicon sizes of qPCR primers.. Resource Type: Text. DOI: https://doi.org/10.22002/gm1q8-v4b87
